# Dermatologic Manifestation of Acro-Ischemia Associated With COVID-19

**DOI:** 10.31486/toj.21.0030

**Published:** 2022

**Authors:** Robert Gumbita, Jason Z. Liu, Rohan Madhu Prasad, Yasser Radwan, Muhammad Nabeel

**Affiliations:** Department of Internal Medicine, Michigan State University, Sparrow Hospital, Lansing, MI

**Keywords:** *Coronavirus*, *COVID-19*, *ischemia*, *SARS-CoV-2*, *skin manifestations*

## Abstract

**Background:** The common dermatologic manifestations seen in patients with coronavirus disease 2019 (COVID-19) include morbilliform, pernio-like, urticarial, macular erythematous, vesicular, and papulosquamous disorders, as well as retiform purpura. Although cases of acro-ischemia have been demonstrated, they are not well studied or reported.

**Case Report:** A 73-year-old male was admitted for acute hypoxic respiratory failure secondary to COVID-19 infection. During the patient's hospital course, his oxygen requirement progressively increased, and he developed painful, violaceous purpura on his right lower extremity digits. The patient was treated with therapeutic doses of enoxaparin and nitroglycerin ointment in the hospital and apixaban on discharge. The patient was lost to follow-up.

**Conclusion:** The multiorgan dysfunction associated with COVID-19 includes dermatologic manifestations. This case illustrates that acro-ischemia can resolve with guideline-based medical treatment.

## INTRODUCTION

Although coronavirus disease 2019 (COVID-19) is known to primarily cause respiratory symptoms, the virus is also associated with multiorgan dysfunction.^1^ Reports indicate that symptomatic dermatologic diseases may arise within a few days.^2-4^ One of the severe dermatologic diseases is distal acro-ischemia, a vasomotor disorder of the extremities that is responsible for distal acute ischemia. At least 12 cases of COVID-19–associated acro-ischemia were published in 2020.^5-9^

## CASE REPORT

**Figure 1.**
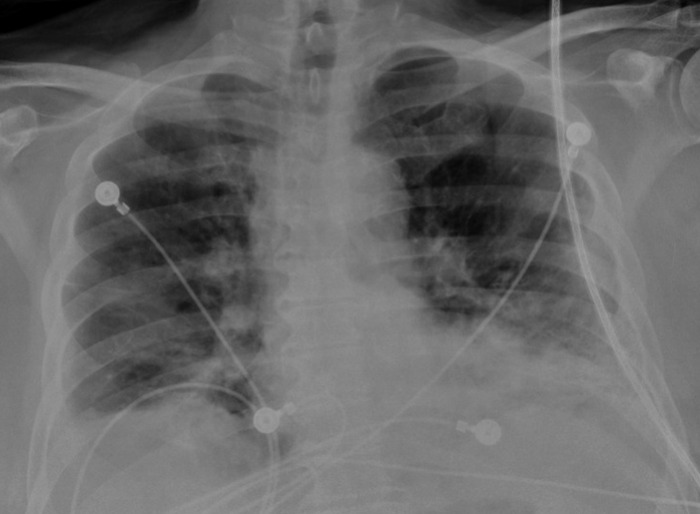
Admission chest x-ray was significant for multifocal bilateral pneumonia and a small left-sided pleural effusion.

A 73-year-old male with a history of non–insulin-dependent type 2 diabetes mellitus, hypertension, and hypothyroidism presented to the emergency department (ED) for shortness of breath. One week prior to presentation, the patient was diagnosed with COVID-19 by polymerase chain reaction nasal swab. Since the diagnosis, shortness of breath, nonproductive cough, and generalized malaise had progressively worsened. On arrival in the ED, the patient was tachypneic and hypoxic, with an oxygen saturation of 75% that required 15 L of 100% oxygen via high-flow nasal cannula. Initial investigations revealed white blood cell count 6.8 × 10^3^/μL (reference range, 4.0-12.0 × 10^3^/μL), C-reactive protein 16.8 mg/dL (reference range, 0-1.0 mg/dL), D-dimer 21.64 mg/L FEU (reference range, 0-0.73 mg/L FEU), lactate dehydrogenase 320 U/L (reference range, 100-225 U/L), ferritin 1,096 ng/mL (reference range, 14-224 ng/mL), and fibrinogen 584 mg/dL (reference range, 150-450 mg/dL). Chest x-ray showed multifocal bilateral pneumonia with a small left-sided pleural effusion (Figure 1). Because of the patient's elevated D-dimer, bilateral lower extremity venous ultrasound was obtained but was negative for deep vein thrombosis. Infectious workup was unremarkable.

The patient was admitted to the intensive care unit for monitoring and management of COVID-19 and was started on hydroxychloroquine (400 mg twice daily on day 1 followed by 200 mg twice daily for 4 days), azithromycin (500 mg daily for 5 days), methylprednisolone (40 mg twice daily for 7 days), and enoxaparin (40 mg daily).

Although the patient was proned intermittently, his volume and fractional inspiration of oxygen still had to be increased. The 15 L of 100% oxygen via high-flow nasal cannula was steadily increased and peaked at 30 L on day 5 of admission. However, the patient did not require intubation.

**Figure 2.**
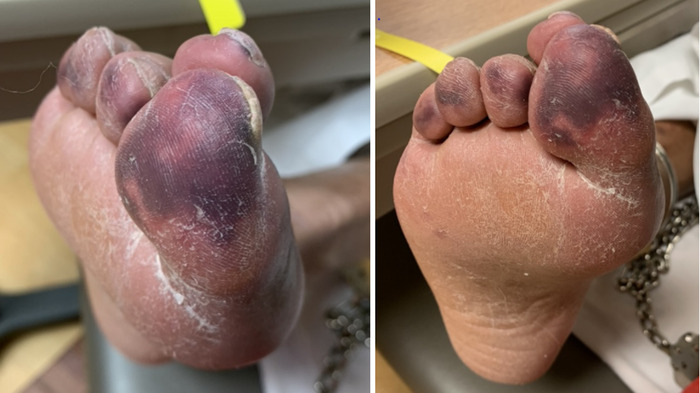
Right lower extremity purpura appeared on day 5 of hospitalization. Areas of discoloration were tender and cool to the touch. Dorsalis pedis and posterior tibial pulses were noted to be 1+ on the right leg.

On day 5, the patient noted acute onset of right foot pain in the distal plantar surface of his great toe and milder pain in all digits. No significant physical examination findings were noted, such as discoloration of the foot or change in pedal pulses. However, the following day, tender, nonblanching, purpuric lesions that were cool to the touch were present on all 5 digits of the right foot. Physical examination of the right leg noted 1+ dorsalis pedis and posterior tibial pulses (Figure 2). These examination changes were persistent for the remainder of the patient's hospital course.

**Figure 3.**
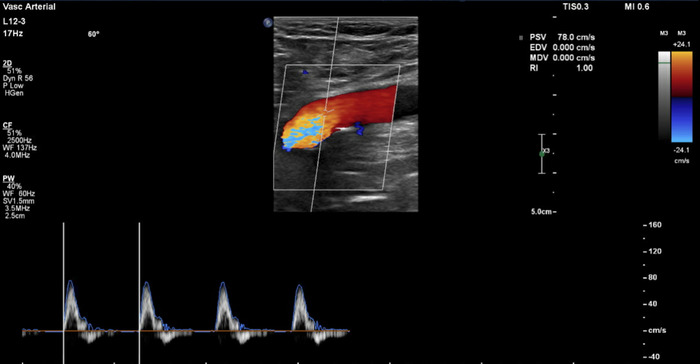
Right lower extremity venous and arterial duplex ultrasound on day 5 of hospitalization depicts near doubling of velocities in the right peroneal artery vs admission, suggesting 50% stenosis. Transition to biphasic waveform suggests mild diffuse atherosclerosis.

On further history, the patient admitted to being a former smoker, reporting that he quit an estimated 40 years prior. He denied any history of chronic obstructive pulmonary disease, cardiovascular disease, coagulopathy, or malignancy. Repeat laboratory workup showed that inflammatory markers were improved, specifically C-reactive protein (2.0 mg/dL), D-dimer (2.13 mg/L FEU), ferritin (503 ng/mL), and fibrinogen (415 mg/dL). Bilateral lower extremity venous and arterial duplex ultrasound showed newly diagnosed 50% stenosis of the right peroneal artery, as well as mild diffuse infrapopliteal atherosclerosis (Figure 3). The finding of infrapopliteal atherosclerosis was illustrated on an arterial duplex ultrasound done 1 year prior to admission.

After these acute changes, the patient was diagnosed with acro-ischemia and was transitioned to a therapeutic dose of enoxaparin 60 mg twice daily on day 5 and then to apixaban 10 mg twice daily on day 13 because of concern for a thromboembolic origin. Nitroglycerin ointment was applied twice daily to the right lower extremity. On day 11, computed tomography angiogram of the chest showed bilateral lower lobe airspace opacities consistent with multifocal, atypical, or viral pneumonia but did not show any evidence of pulmonary embolism.

The patient was discharged home on day 15 with instructions to use 5 L of oxygen during exertion, to complete a 3-month course of apixaban, and to apply nitroglycerin ointment to his right foot. The patient was lost to follow-up, so his outcome is unknown.

## DISCUSSION

The pathology associated with severe acute respiratory syndrome coronavirus 2 (SARS-CoV-2), the virus that causes COVID-19, is still being determined. An exaggerated immune response that creates a significant risk of causing damage to the lungs, heart, kidneys, neurologic system, and gastrointestinal system has been proposed.^1^ In addition, various cases have described dermatologic manifestations of the virus that include morbilliform, pernio-like, urticarial, macular erythematous, vesicular, and papulosquamous disorders, as well as retiform purpura***.***^10^ However, the pathogenesis of these manifestations in relation to SARS-CoV-2 remains elusive.

SARS-CoV-2 targets angiotensin-converting enzyme 2 (ACE2) receptors^11^ that are expressed on lung epithelial cells, macrophages, enterocytes, and other target cells. Activation of these receptors leads to an inflammatory immune response that may be responsible for the lung and other organ damage associated with the virus. These ACE2 receptors are also present in arterial and venous endothelial cells, as well as in the epidermal basal layer.^12^ Endotheliitis has been proposed as a possible cause of impaired microcirculatory and dermatologic sequelae associated with COVID-19.^12,13^

In severe cases of COVID-19, a high incidence of hypercoagulability has been noted that manifests as either deep vein thrombosis or pulmonary embolism.^14^ Although our patient did not require intubation or vasopressors, his oxygen requirements acutely increased. Given the patient's increased inflammatory markers of C-reactive protein, D-dimer, and ferritin, along with the diminished pulses in his right lower extremity, microemboli traveling to the vasculature of the distal right lower extremity may have contributed to his acro-ischemia. Studies have reported that patients with acro-ischemia typically worsen throughout their hospital course and develop significant complications, specifically cerebral infarcts and death.^6,9,14-18^ The differential diagnoses for an adult developing purpuric lesions and acro-ischemia include microvascular occlusion syndromes, arterial emboli, hypercoagulable disorders, and vasculitis.^19^ Our case demonstrates that COVID-19 should also be considered and ruled out. The acro-ischemia was attributed to COVID-19 because of the temporal onset of the disease, the patient's elevated D-dimer, and arterial Doppler findings of right peroneal artery stenosis and mild diffuse infrapopliteal atherosclerosis.

## CONCLUSION

COVID-19 is commonly associated with damage to the lungs, heart, and kidneys. However, physicians should be aware that COVID-19 can also cause dermatologic manifestations. This case highlights the presentation of acro-ischemia in a critically ill patient infected with COVID-19 that responded well to guideline-based medical treatment.

## Notes

*This article meets the Accreditation Council for Graduate Medical Education and the American Board of Medical Specialties Maintenance of Certification competencies for Patient Care and Medical Knowledge.*
